# ‘Pagbangon at Pag‐Asa’ (Resurgence and Hope): A Qualitative Study of the Lived Experiences of People With Stroke and Household Carers in the Philippines

**DOI:** 10.1111/hex.70537

**Published:** 2025-12-24

**Authors:** Sarah Ann Buckingham, Elda Anota, Maria Mercedes Barba, Vergil Boac, Myrna Estrada, Lorraine Faeldon, Bridie Kent, Fiona Leggat, Roy Francis Navea, Aira Patrice Rueda Ong, Nena Marie Santos, Annah Teves, Paula Melizza Valera, Fiona Jones, the TULAY project team

**Affiliations:** ^1^ School of Nursing and Midwifery, Faculty of Health University of Plymouth Plymouth Devon UK; ^2^ Evelyn D. Ang ‐ Institute of Biomedical Engineering and Health Technologies De La Salle University Manila The Philippines; ^3^ Population Health Research Institute, School of Health and Medical Sciences, City St. George's University of London London UK; ^4^ Bridges Self‐Management Limited London UK

**Keywords:** interviews, lived experiences, low‐middle‐income country, Philippines, qualitative, stroke, visual elicitation

## Abstract

**Background:**

Stroke is a leading cause of disability and mortality in the Philippines, where access to formal rehabilitation services is limited. There is a lack of research on the lived experiences of people with stroke and their household carers across diverse urban and rural settings.

**Objective:**

To explore the experiences, challenges and support needs of people with stroke and their household carers throughout the Philippines, and to inform the co‐design of a community‐based stroke support programme.

**Methods:**

A descriptive qualitative design was used. Semi‐structured interviews were conducted with 24 people with stroke and 20 household carers across six sites in Luzon, Visayas and Mindanao. Interviews were enriched by auto‐photography and visual elicitation. Data were analysed thematically using an interpretivist approach, through collaborative analysis by UK‐ and Philippine‐based researchers.

**Results:**

Four key themes were identified: (1) *Multidimensional burden of stroke for people with stroke and household carers*, including physical, psychological, social and financial impacts; (2) *Cultural management and coping practices*, emphasising the central role of family, use of traditional therapies and adaptive strategies, rooted in cultural values such as ‘pagtitiis’ (resilience and endurance) and ‘utang na loob’ (reciprocal obligation); (3) *Knowledge and awareness of stroke and rehabilitation*, revealing significant gaps and reliance on personal experience and informal information sources; and (4) *Stroke care and rehabilitation needs, challenges, and recommendations*, showing limited service availability and geographical and financial barriers, particularly in rural areas, alongside a strong desire for accessible, community‐based support.

**Conclusion:**

Culturally relevant, gender‐sensitive and accessible community‐based stroke support and education programmes are urgently needed in the Philippines to address unmet needs and inequities in immediate and longer‐term care.

**Patient or Public Contribution:**

A PPIE group consisting of people with stroke and other physical disabilities and carers reviewed the study protocol, interview topic guides and participant‐facing documents and provided feedback on initial themes.

## 1 Introduction

Stroke is a medical emergency and a leading cause of death and disability worldwide [1]. In the Philippines, it is the second leading cause of mortality, accounting for approximately 14% of total deaths [2]. Stroke survivors frequently experience a range of motor, sensory, cognitive and communication impairments, which significantly impact daily functioning and quality of life [3].

In low‐middle‐income countries (LMICs), this burden is compounded by systemic healthcare challenges related to affordability and availability of services [4, 5, 6]. In the Philippines, most healthcare costs are paid out‐of‐pocket, limiting access for lower‐income groups [7, 8, 9]. Stroke units and rehabilitation services are concentrated in urban areas, with rural and underserved areas facing major gaps in specialist care [9, 10, 11]. Community‐based care is underdeveloped, and there is a nationwide shortage of trained rehabilitation professionals [7, 11].

There is a critical gap in understanding the broader lived experiences and unmet needs of people with stroke and their household (informal and unpaid) carers in LMICs. While some recent qualitative studies have begun to explore aspects of stroke care in the Philippines, these have been limited in scope and setting, focusing on practical experiences of caregiving among small samples of caregivers in urban Cebu [12, 13, 14].

This paper presents the first multiregional qualitative study of stroke experiences in the Philippines, conducted as part of the 4‐year TULAY project (Tulong, Ugnayan ng Lingap At gabaY—help, compassion and guidance). The aims of the project are to map current services, uncover participant priorities, and co‐design a context‐appropriate, community‐based stroke support programme that meets the needs of people with stroke, their household carers and professional care providers. Building on a recent national survey (completed May 2024, manuscripts currently under review) that captured the experiences of these groups in relation to stroke services, this qualitative study was undertaken to deepen and contextualise those findings. Focusing on the perspectives of people with stroke and their household carers (care provider findings will be reported separately), the study aimed to explore their lived experiences and unmet needs and to develop a richer understanding of the nature and quality of stroke rehabilitation and care in both urban and rural settings.

## 2 Materials and Methods

### 2.1 Overview and Design

A descriptive qualitative design within an interpretivist paradigm [15] was used. Data were collected through semi‐structured interviews, complemented by auto‐photography and visual elicitation. A full description of the methods is published elsewhere [16]. The Consolidated Criteria for Reporting Qualitative Research (COREQ) checklist [17] was followed (Supporting File 1).

### 2.2 Participants and Recruitment

Inclusion criteria for people with stroke were adults aged 20 years and above, including people with aphasia but not lacking capacity. Household carers were aged 18 years and above, including relatives, friends or neighbours who supported a person with stroke. Carers bereaved within the last 5 years were also included.

Participants were purposively sampled from a national survey database generated by the TULAY project, which included 498 people with stroke and 487 household carers across the Philippines, of whom 452 (90.8%) and 443 (91.0%), respectively, had expressed willingness to participate in follow‐up interviews. Sampling aimed for diversity across different localities (including six cities/municipalities within five regions of Luzon, Visayas and Mindanao), urban and rural locations, and socio‐economic level based on self‐reported and municipal‐level indicators. Stroke severity and health status were also considered. As some individuals were in remote locations that were not feasible to reach and/or did not respond to researchers' contacts, additional recruitment support was provided by city and rural health units. These units used their own databases to identify and recommend individuals within the selected areas who were accessible and willing to be interviewed.

### 2.3 Procedures

#### 2.3.1 Informed Consent

Selected participants received a paper or digital invitation letter, information sheet, and consent form. Consent items included interview participation, audio and video recording, and use of media in the TULAY programme. Due to geographic spread, a flexible approach was used with verbal, written or same‐day consent as appropriate. In all cases, the study purpose was explained, and consent was reconfirmed on the interview day.

For participants with aphasia or low literacy, capacity was assessed by a trained researcher based on understanding, retention and ability to communicate a voluntary decision. Simplified language, visual cues and extra time were used as needed, with family members or carers assisting with communication but not providing consent. All researchers received training in supported communication and ethical consent procedures.

#### 2.3.2 Interviews

Semi‐structured interviews were conducted in person between June and August 2024. The aim was to recruit 10 participants in total per locality (including people with stroke, household carers and care providers), and this sample size was deemed sufficient as data saturation was achieved, with no new themes emerging during the final interviews. All interviews took place in participants' homes, in their preferred language.

Ten trained researchers in the Philippines, including men and women of varied ages, all with academic backgrounds and some qualified healthcare professionals, carried out the interviews. One researcher led each interview while another handled recording and note‐taking. All interviewers were part of the TULAY project and had a professional or personal interest in stroke care. Interviewers and participants had no prior relationship, but the local knowledge of the interviewers helped to foster rapport and open communication.

Topic guides were co‐developed with the Patient and Public Involvement and Engagement (PPIE) group and informed by earlier project phases. Topics included experiences of stroke, rehabilitation, barriers and enablers to accessing care and managing stroke, and service improvement suggestions (Supporting Files 2 and 3). The topic guides were piloted with one person with stroke and one household carer. Interviews followed a flexible structure with open‐ended questions, probing and reflection to encourage rich responses. The interviews were audio‐ or video‐recorded with participants' consent, and researchers took detailed field notes to record contextual observations and reflections during and after the interviews.

#### 2.3.3 Auto‐Photography and Visual Elicitation

People with stroke and household carers were invited to optionally share photographs or videos (digital or non‐digital) that illustrated their experiences, recovery and life after stroke. Alternatively, participants could draw or show an object, which was then photographed by the interviewer. The visual media served as prompts for discussion, helped to build rapport, supported communication (especially for those with aphasia) and deepened understanding across cultural or language differences. These options were included to enhance the richness and trustworthiness of the data [18, 19].

### 2.4 Data Analysis

All interviews were transcribed and, where necessary, translated into English, with care to preserve key Filipino phrases and cultural context. The researchers who conducted the interviews carried out transcription and translation, ensuring accurate interpretation through their familiarity with the interviews and local setting.

Thematic analysis was undertaken using Braun and Clarke's methods [20, 21], with a combination of inductive and deductive analyses. Analysis was conducted collaboratively by UK and Philippine researchers, supported by NVivo 14 and the Collaboration Cloud [22]. Six researchers coded the data in NVivo, and the wider team contributed to theme development. NVivo queries were used to explore differences by age, gender, socio‐economic status, region, and urban and rural areas. Thematic concept maps were developed by the research team and visually presented using Xmind AI [23].

Although the main purpose of the photographs and videos was to stimulate discussion in the interviews, they were organised and coded with short descriptive notes. This process was guided by visual analytic methods, including numbering, counting, coding using participants' own descriptions and organising into broad and specific categories [18, 24, 25].

Multiple strategies were used to ensure rigour, trustworthiness and sensitivity to cultural context. Topic guides with defined terms and the use of both visual and written data enhanced consistency. A sample of transcripts (*n* = 4) was jointly coded by UK and Philippine researchers, with high overall inter‐coder agreement (approximately 86%). All researchers documented observations and reflexive notes during coding, and summaries were developed collaboratively. Weekly analysis meetings facilitated reflection, discussion and peer debriefing. Member reflections were undertaken by sharing summaries of the findings with four people with stroke and two household carers, who affirmed the relevance and resonance of the identified themes and contributed interpretive insights. The findings were also shared with the PPIE group. A full audit trail, including coding decisions and the analysis process, was maintained in NVivo.

Interview participants were given an alphanumeric identifier (PS = Person with Stroke; HC = Household Carer). Demographic data are not included with individual quotations to protect participants' anonymity.

## 3 Results

### 3.1 Participant Characteristics

Twenty‐four people with stroke and twenty household carers were interviewed, with interviews lasting from 18 to 88 min. Of the 43 interviews (including one joint interview), 23 were conducted in Tagalog and 20 in Cebuano/Bisaya, two widely spoken languages of the Philippines.

Participant demographics are provided in Table 1. Household carers tended to be younger than the person with stroke and more often female. Most participants lived in rural areas and were of low socio‐economic status, though a range of regions and backgrounds were represented.

**Table 1:** Demographics and characteristics of interview participants.

Variable	People with stroke (*N* = 24)	Household carers (*N* = 20)
Age (years)		
Mean (SD)	59.0 (10.3)	49.8 (8.7)
Range	33–77	26–60
Gender, *n* (%)		
Male	14 (58)	4 (20)
Female	10 (42)	16 (80)
Ethnicity, *n* (%)		
Tagalog	5 (21)	3 (15)
Illonggo/Karay‐a	4 (17)	5 (25)
Cebuano/Binisaya/Bisaya/Bol anon	12 (50)	9 (45)
Unknown	3 (13)	3 (15)
Locality/region, *n* (%)		
Las Piñas (NCR, Luzon)	4 (17)	3 (15)
Carmona (Calabarzon, Luzon)	4 (17)	3 (15)
Culasi (Region 6, Western Visayas)	4 (17)	5 (25)
Batuan (Region 7, Central Visayas)	3 (13)	3 (15)
Initao (Region 10, Northern Mindanao)	5 (21)	3 (15)
Medina (Region 10, Northern Mindanao)	4 (17)	3 (15)
Urban or rural residence, *n* (%)		
Urban	7 (29)	6 (30)
Rural	17 (71)	14 (70)
Socio‐economic status, *n* (%)		
Low	21 (88)	17 (85)
Middle/high	3 (13)	3 (15)
Time since stroke (years)		N/A
Mean (SD)	5.3 (4.1)	
Range	0.3–15	
Previous stroke, *n* (%)		N/A
Yes	4 (17)	
No	20 (83)	
Communication difficulties or aphasia, *n* (%)		N/A
Yes	8 (33)	
No	16 (67)	
Relation to person with stroke, *n* (%)	N/A	
Spouse or partner		10 (50)
Brother or sister		5 (25)
Son or daughter		5 (25)

*Note:* Percentages may not total 100 due to rounding. Urban/rural residence was self‐reported in a questionnaire completed prior to participation. Classification of socio‐economic status was based on self‐reported data on education and income; where these data were not available, regional indicators of poverty incidence were used as an estimate, that is, lower than the national average = middle/high socio‐economic status, higher than the national average = low socio‐economic status (as defined by the Philippine Statistics Authority [Philippine Statistics Authority. Philippine Statistics in Brief {First Quarter 2024}. Quezon City, Philippines. Accessed May 20, 2024, https://library.psa.gov.ph/cgi-bin/koha/opac-retrieve-file.pl?id=70e5accd94be90f3bf7664912433a3a9]).

Abbreviations: N/A = Non‐Applicable, SD = Standard Deviation.

### 3.2 Results of Visual Elicitation

**Figure 1:**
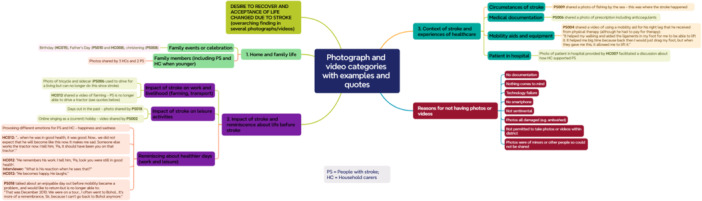
Results of visual elicitation.

Eleven of the forty‐four participants (seven people with stroke and four household carers) shared one or more photographs or videos. As this represented only a small proportion of participants, and the visual elicitation was intended to provide complementary insights into participants' experiences rather than form a core component of the thematic analysis, the visual findings are presented separately (Figure 1). Participants' reasons for being unable to share a photograph or video are also shown in Figure 1.

The data were organised into three main categories. These were home and family life (including family members and family events); impact of stroke and reminiscence about life before stroke (including work and leisure); and context of stroke and experiences of healthcare (from time of stroke to hospital admission to therapy received). Overall, the photographs and videos represented a strong desire to recover but also recognition and acceptance (albeit reluctant) that their lives had changed due to the stroke. The visual reminders of life before stroke provoked contrasting emotions of happiness and sadness.

### 3.3 Interview Themes

**Figure 2:**
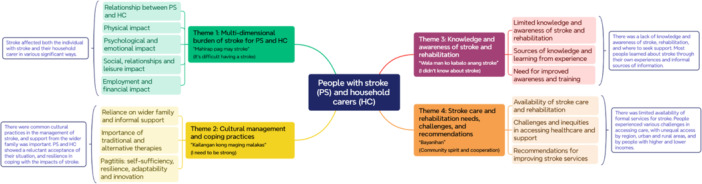
Concept map of interview themes with summaries.

Analysis of the interviews resulted in six initial categories with a total of 97 codes. These were developed into four key themes, each with several sub‐themes (Figure 2).

#### 3.3.1 Theme 1 Multidimensional Burden of Stroke for People With Stroke and Household Carers: ‘Mahirap Pag May Stroke’ (It's Difficult Having a Stroke)

##### 3.3.1.1 Relationship Between Person With Stroke and Household Carer

People with stroke relied heavily on family members for daily care, including spouses, children and siblings. Caregiving was perceived as a duty or expectation, and the role often fell to women, who balanced these responsibilities with household duties and employment. Younger carers faced additional challenges juggling studies or early careers. Caregiving durations ranged from a few months to 20 years.

Many people with stroke and household carers were coping with other health conditions, including diabetes, heart disease, arthritis and sensory impairments. Some developed co‐dependent relationships, supporting each other through illness.

In general, people with stroke expressed deep gratitude for their carers:

***Interviewer:*** Where do you draw strength?

***PS008:*** My siblings! When there are people who understand me, that's what I hold on to … if it wasn't for their help I wouldn't be alive.

While most relationships were supportive, some were strained by stress, frustration and communication difficulties. Some household carers described how personality changes, stubbornness and irritability in the person with stroke led to arguments and made caregiving more challenging:

It became more difficult upon coming home. Taking care of someone is hard. And then now, the person I am taking care of is being stubborn. Even refusing to eat…. When I get frustrated, I just go to the room so we won't argue. Sometimes the stubbornness gets into my nerves.

HC001

Shifts in family dynamics added to this strain. Some carers noted that their loved one could no longer fulfil their previous head‐of‐household role, with spouses or children becoming the main breadwinners or providing care similar to that given to a child:

I really took care of him … like a baby.

HC014

He needs to be hugged like a child.

HC012

##### 3.3.1.2 Physical Impact

Many people with stroke experienced significant mobility issues, including one‐sided weakness, difficulty walking, transferring and performing daily tasks like bathing and dressing. Some required complete assistance, while others used mobility aids. Pain, balance problems, and cognitive and communication difficulties were also reported. For example:

I didn't think that having this kind of stroke would be very uncomfortable, with the limited movements due to the impairment on the left side. Sometimes it's unbearable, as I cannot do anything.

PS003

Some household carers reported exhaustion due to the physical demands of caregiving, such as lifting and assisting with mobility. *HC011* described the toll this took:

I was so exhausted, I thought I would be the first to die. I had to feed her, change her clothes, everything.

HC011

Physical impact varied widely depending on stroke severity and recovery. A few participants reported minimal disruption, continuing with physically demanding tasks, for example:

Things are still the same, but here, whatever I can do, I'll work on it…. Yes, I built the fence around here. I also hauled the bamboo for it, I dragged it.

PS022

##### 3.3.1.3 Psychological and Emotional Impact

People with stroke frequently experience emotional distress, including depression, self‐pity and feelings of helplessness. Some reported struggling with frustration, mood swings, irritability and anger:

I always get easily irritated. Yes, I was angry at God, asking why this happened to me?

PS020

A sense of loss was expressed by many—the loss of their ability to work, to perform daily activities independently, or the loss of their previous lifestyle:

He seems mentally burdened, frustrated that he can't do what he used to. That's likely why he gets irritable.

HC018

Although most people appeared to be coping and showed resilience, some felt hopeless, and one even considered ending their life to avoid burdening their family. Unhealthy coping mechanisms were used by a few participants, including high alcohol consumption and eating unhealthy snacks.

Caregivers also reported emotional strain, including sadness, anxiety and fatigue, especially when managing challenging behaviours. The deep emotional connection and empathy between people with stroke and their family members was clear. For example, one carer talked about the impact of their mother's stroke on their sibling:

My younger sibling would say, ‘Don't cry, Ma, because we feel like crying too when you cry’…. She cries because of what happened to her, that she became paralysed.

HC016

##### 3.3.1.4 Social, Relationships and Leisure Impact

Both people with stroke and household carers experienced social isolation. People with stroke often withdrew from friends, extended family and the community, due to mobility limitations, or embarrassment or shame (‘hiya’) about their condition:

When I could walk, many people would call out to me. Now that I just sit around, no one calls me anymore. I just stay at home.

PS019

They [the neighbours] gossip…. I mostly just stay at home.

PS020

Household carers had their social lives severely restricted due to a lack of free time, limited finances due to medical costs, or fear of leaving their loved one at home alone:

I could not really go out now, Sir. Since their stroke.

HC015

Leisure activities were frequently sacrificed for people with stroke and their carers.

Family relationships could become strained—not only between the person with stroke and their carer, but also among other family members. One carer shared that her marital relationship had suffered due to difficulties balancing her role as carer for her mother with looking after her own home and family:

Sometimes we fought, but I try to understand him [husband]. Because I can't always take care of our home since I'm often here.

HC016

##### 3.3.1.5 Employment and Financial Impact

Almost all the people with stroke who were previously employed faced challenges in returning to work after their stroke. This was most evident for those in manual jobs such as fishing, farming, carpentry and factory work, which are common occupations in the Philippines:

There were no jobs that would take me. Work in land, as a labourer, they wouldn't take me because I couldn't move like before.

PS016

Many household carers also had to leave their jobs or reduce working hours, leading to significant financial strain. Medical costs (medications, hospital stays and therapy) placed additional economic burdens on families, with many struggling to make ends meet. Some families were forced to sell property or had to rely on limited external support:

It's very difficult particularly with the financial aspect. Because we ended up pawning our mother's properties, we also sold our land for her.

HC015

When he had a stroke, it was all over. Everything was cut off. All the dreams…. It's difficult, I don't understand where we can get financial support.

HC005

#### 3.3.2 Theme 2 Cultural Management and Coping Practices: ‘Kailangan Kong Maging Malakas’ (I Need to Be Strong)

##### 3.3.2.1 Reliance on Wider Family and Informal Support

In addition to the household carer, many people with stroke depended on other family members for physical and personal care, help with household chores, financial support for living and medical expenses, and emotional and social support. Some also assisted with medication management and transport to medical appointments. Support typically came from nuclear and extended family living in the same household or nearby, but there were exceptions, for example, one participant received financial support from a child working abroad:

I only have this small coconut farm. And my child who's working outside the country by sending money for her children that lives here, in which I can be included in the consumption. Because my small coconut farm can't provide enough for all of us here, especially since I have so many grandchildren.

PS021

However, not all families were able to provide adequate assistance, and a small number of people with stroke lived alone. Others were reluctant to ask for help as they were embarrassed about their condition, afraid of being a burden, or simply wanted to maintain their independence.

Two participants had pets that they felt helped to reduce their loneliness and isolation. *PS001* shared:

The reason why I adopted a dog was to have someone to talk to, someone to watch over me. Because some people come visit but go home after a day.

PS001

Support for household carers was variable and inconsistent, although in some cases, extended family members and barangay health workers (BHWs) helped to provide some relief from caregiving duties, and some received financial or emotional support from family members or friends.

##### 3.3.2.2 Importance of Traditional and Alternative Therapies

Traditional practices such as ‘hilot’ (physical and spiritual massage), herbal remedies (e.g., medicines, liniments and oils such as kalabo and tuba‐tuba/jatropha oils), and faith healing were widely used. These were often provided by family members and held greater importance in rural areas where healthcare services were less available and accessible. Such therapies were perceived as an affordable and sometimes better alternative to hospital treatment and rehabilitation. As one carer explained:

At home, his blood pressure decreased. I gave him herbal medicine, therapy, traditional massage. He became better than in the hospital, you know, because there are many sick people and the place is hot.

HC011

##### 3.3.2.3 ‘Pagtitiis’: Self‐Sufficiency, Resilience, Adaptability and Innovation

A unique Filipino trait reflected as a universal theme through the interviews was ‘pagtitiis’, a term meaning endurance, patience and the silent bearing of hardship. Despite experiencing pain and significant challenges (Theme 1), people with stroke and their carers rarely complained, instead managing their difficulties independently. This was underpinned by a mindset of ‘bahala na’ (‘come what may’, or ‘leave it to God or fate’), reflecting cultural values of acceptance and faith:

I need to be strong; I need to endure.

PS008

**Figure 3:**
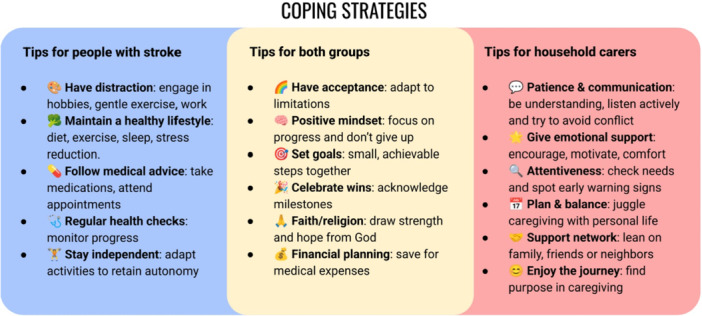
Participants' coping strategies and tips.

This demonstrated strength and resilience in managing life after stroke, and participants shared practical coping strategies for others in similar situations (Figure 3).

There were excellent examples of resourcefulness, adaptability and innovation. Some people sold personal belongings or cultivated food at home to offset living costs and medical expenses. Extended family members and sometimes the neighbourhood community came together to support the person with stroke. Various creative solutions and adaptations to address mobility challenges and limitations in daily life were implemented using locally available materials. These included bamboo handrails and rope to support mobility, walking canes made from guava branches, a wooden chair for transport, and a tool for collecting firewood, as described by one participant:

I use a cane with a knife blade attached to the end. Then I just connect it to a bamboo, where I put a string on it, and that is where I tie the firewood.

PS022

#### 3.3.3 Theme 3 Knowledge and Awareness of Stroke and Rehabilitation: ‘Wala Man Ko Kabalo Anang Stroke’ (I Didn't Know About Stroke)

##### 3.3.3.1 Limited Knowledge and Awareness of Stroke and Rehabilitation

In general, people with stroke and household carers lacked awareness of stroke causes, symptoms, treatment and recovery options.

***PS021:*** I don't know about stroke because those who've had strokes here live far away.

***Interviewer:*** So you don't know what a stroke is, Ma'am?

***PS021:*** No.

***Interviewer:*** Do you know what should be done to recover quickly after a stroke?

***PS021:*** I don't know anything about that either.

***Interviewer:*** Did anyone teach you about it or talk to you about it?

***PS021:*** No.

Misconceptions about stroke were prevalent, for example, confusing it with heatstroke and believing it only affected older people:

***PS023:*** I suppose you wouldn't get a stroke if you were still young, right?

***Interviewer:*** Oh, there are cases, Sir! There are young people who suffer from strokes too.

***PS023:*** Ah, but they can handle it—it's mostly heatstroke … or maybe just a mild stroke!

This lack of knowledge about stroke and its symptoms contributed to delayed or inappropriate health‐seeking behaviours, as many individuals were unaware of the condition's severity and urgency of seeking medical treatment. For example, *PS016* recounted:

I told them [medical staff], ‘It's farfetched that I had a mild stroke when my body feels healthy.’

PS016

There was limited understanding and varying perceptions of the value of rehabilitation. While some participants viewed it as ‘essential’, others questioned its usefulness:

If I hadn't gone to rehab, I wouldn't be able to walk.

PS019

I didn't follow through…. I didn't go back to the schedule because it's just exercise, we can do it at home.

PS018

Traditional therapies such as massage were often regarded as equivalent to rehabilitation, and most participants were unaware of support options beyond their families and immediate neighbourhoods.

##### 3.3.3.2 Sources of Knowledge and Learning From Experience

Stroke knowledge was usually acquired informally, through personal experience, online resources (primarily social media including Facebook and YouTube), and advice from family, friends and neighbours. A small number of people had received some advice or guidance from healthcare professionals, but none had received formal education on rehabilitation or long‐term management.

Both people with stroke and household carers relied greatly on their own experience for managing stroke. This included experience gained over time or from a previous stroke, experience with other conditions (such as massaging sprains), or their instinct. Household carers gradually adapted to the caring role and developed coping strategies through trial and error, relying on their instincts and support and advice from family members:

At first, I had none [knowledge]. We didn't know anything about it, but my mother knew that when this happens, you have to massage the patient so that the nerves/veins would come alive again. That's what I did. I remember what my mom said.

HC002

##### 3.3.3.3 Need for Improved Awareness and Training

The interviews indicated a strong need and desire for educational programmes on stroke. Preferred content included understanding stroke; how to prepare for or prevent further strokes; home‐based rehabilitation (e.g., exercises and massage); caregiving techniques (e.g., mobility support and medication management); and accessing support (physical, mental, spiritual and financial).

Preferences for delivery mode and format varied, although many participants favoured visual content, videos and practical demonstrations (e.g., exercises) delivered by healthcare professionals. Participants with adequate internet access wanted some resources to be online, whereas others lacked the necessary technology.

Preferences for one‐to‐one versus group training were influenced by personal circumstances such as the ability to travel and level of sociability. For example:

I prefer one‐on‐one…. I'm not very social.

PS008

It's better to have company. It's lonely when we're alone.

HC011

One carer summarised the need for more practical, hands‐on training to reduce their uncertainty about how to respond in an emergency situation:

It would be great if there were more training because I know it's not enough. It's different when you're hands‐on versus just being oriented briefly…. I'd like to learn more about what to do for someone who had a stroke, especially if they can't walk well…. What are the proper steps to take? If he has another stroke, what should I do to avoid panicking? What should I give him?

HC012

#### 3.3.4 Theme 4 Stroke Care and Rehabilitation Needs, Challenges and Recommendations: ‘Bayanihan’ (Community Spirit and Cooperation)

##### 3.3.4.1 Availability of Stroke Care and Rehabilitation

The interviews indicated a widespread lack of formal stroke services, although availability varied by location, with more hospitals and rehabilitation centres in northern and urban areas. In most places, professional rehabilitation was limited and required travel. While some participants received home visits from BHWs for basic health monitoring, home‐ and community‐based rehabilitation programmes were scarce. Many relied on informal rehabilitation (e.g., exercises and massage) provided by family members, and others received no rehabilitation at all. For example, one household carer talked about living in rural Medina, where there were no local services and home rehabilitation was not available:

The therapist wouldn't do home service. We were ready to pay the cost (₱4000), but they wouldn't come to the house…. The therapist had a clinic at their house, so patients had to go there, which was very inconvenient.

HC017

##### 3.3.4.2 Challenges and Inequities in Accessing Healthcare and Support

Beyond service availability, people with stroke faced barriers to care due to financial limitations, with medical expenses typically paid out‐of‐pocket. Some had received one‐time government aid through elected officials (e.g., mayors), Malasakit Centres (one‐stop shops for medical and financial assistance), Medical Assistance for Indigent Patients (MAIP), or Aid to Individuals in Crisis Situations (AICS), but this support was minimal and did not cover ongoing rehabilitation needs. Persons With Disability (PWD) ID cards provided discounts on essential goods and services, but these were often insufficient. Administrative barriers in accessing government aid, lack of trust in government systems and limited information about available support were also mentioned. As one household carer stated:

*What we really want is for him to have therapy. But we don't have the money. We're really short financially*.

HC005

While free medications were sometimes available at city and rural health centres, there were frequent stockouts, and access to essential medication appeared to depend on individual persistence and personal advocacy:

They always run out of Losartan, so I just buy it myself…. I complained, and only then did they give me medicine.

PS004

Geographical barriers including distance from healthcare facilities and difficult terrains (e.g., mountainous areas) were commonly reported. These were compounded by a lack of transportation, including a shortage of ambulances.

Although issues were reported in all regions and demographic groups, greater challenges were experienced in the southern, rural and geographically isolated regions and by people of lower socio‐economic status. Public healthcare facilities were often under‐resourced, while private healthcare was unaffordable for many.

Despite these challenges, ‘Bayanihan’—the Filipino spirit of community cooperation—was evident. Traditional ‘Paluwagan’ groups, informal savings collectives based on mutual trust and shared financial responsibility, were formed to help with medical costs. In some cases, neighbours and local rescue teams provided vital support, including transportation to the hospital.

##### 3.3.4.3 Recommendations for Improving Stroke Services

Many participants found it difficult to articulate suggestions for improving stroke services. This seemed to stem from limited knowledge, uncertainty regarding their own needs, and cultural factors, including deference to authority and a tendency to accept what is provided rather than advocate for additional support.

However, when prompted by the interviewers, several recommendations were made, including financial support, help to return to work, improved availability and access to local services, education and training programmes, and community support groups (Table 2). ‘Bayanihan’ was again an overarching theme, with participants calling for government, health services and communities to work together to improve the lives of people with stroke—building a ‘community of caretakers’.

**Table 2:** Participants' recommendations for improving stroke services.

Recommendation	Example quote(s)
Improved financial support for medical expenses, and prioritisation of stroke by the government	‘…our voice isn't that strong for us to be prioritised. We go through, of course, challenges, and sometimes we even get rejected. So I hope … that's all we're asking for from TULAY … to help us with this’. **(HC003)**
Improved availability of local stroke rehabilitation facilities and programmes	‘Yes, they offer therapy there, those kinds of services, but there's nothing like that here. They really should establish something like that’. **(HC011)** ‘I really want a facility even if it's only accessible via our municipality. Even one [facility] is fine, as long as there is one to go to’. **(HC007)**
Local diagnostic facilities (e.g., CT scans)	‘I hope there's something like that [CT Scan machine] here in Initao, so we don't have to go to Cagayan anymore. It's tiring to travel there, and it's an additional cost for the fare’. **(HC016)**
Better distribution of free medication and mobility equipment in rural health units (to overcome logistical and financial challenges to access)	‘As long as I have medicine, I'm okay. No more problems’. **(PS016)** ‘Sometimes people need wheelchairs, but they don't have one’. **(HC003)**
More home visits for health monitoring by healthcare professionals	‘He [person with stroke] wants the doctor to visit here … he can walk but it is difficult for him’. **(HC019)**
Provision of livelihood activities and help to return to work (to promote activity, independence, financial stability and self‐esteem)	‘It would be better if there were more activities … work‐related activities, like gardening … so that good thoughts or talents can come back’. **(PS022)**
Support groups for people with stroke and household carers (for sharing experiences and mutual support)	‘I'd like to know their story too. Sharing [with other stroke survivors] … so we would know about our current condition … I'll learn what helps’. **(PS015)**
Stroke awareness, education and training for healthcare professionals, people with stroke and their families (including prevention and management of stroke)	‘It [training] would really help us in taking care of our father…. I would like to join programmes like that, to learn’. **(HC018)**

## 4 Discussion

### 4.1 Discussion and Implications of Findings

This study was the first to provide an in‐depth insight into the lived experiences of people with stroke and their household carers in the Philippines. The four main themes will be discussed in turn, followed by implications for policy and practice.

#### 4.1.1 Multidimensional Burden of Stroke for People With Stroke and Household Carers

Stroke impacted multiple aspects of life for both people with stroke and their carers. Physical impairments resulting from stroke were common, and household carers experienced fatigue and exhaustion. Emotional strain was pervasive, with frequent reports of stress, anxiety and depression, and, in some cases, helplessness and hopelessness. A sense of loss was evident, and reminiscing about lives before stroke evoked mixed emotions of happiness and sadness. Stroke also disrupted family dynamics, strained relationships and contributed to social isolation.

These findings are consistent with the global literature on stroke. Feelings of loss, helplessness and social isolation for people with stroke have been reported in prior qualitative studies in various countries [26, 27, 28]. Similarly, physical, psychological, emotional and social burdens on family caregivers of people with stroke have been reported in other LMICs [6, 28].

The financial burden was severe; most people with stroke were unable to return to work, and many household carers reduced hours or left employment. Selling property or land to cover medical expenses was common. In LMICs, such financial strains are exacerbated by limited healthcare infrastructure [6]. Despite these challenges, participants expressed a strong desire to regain livelihood and purpose.

#### 4.1.2 Cultural Management and Coping Practices

Stroke care was primarily managed within the home, typically by female relatives in large, multigenerational households; this reflects traditional Filipino family structures and gender roles. In such households, older people are often cared for by their adult children [29], while women are expected to manage the household and finances [30, 31]. Family caregiving is a deeply rooted cultural norm in the Philippines, as in many Asian countries and LMICs [6, 32, 33], grounded in values such as ‘filial piety’, a moral duty to care for elders [34, 35], and ‘utang na loob’, a sense of reciprocal or social obligation [36, 37]. Failure to fulfil caregiving or financial responsibilities may be viewed as shameful or ‘nakakahiya’ [38], reinforcing reliance on family or extended networks rather than external health or social services.

Despite the significant burdens of living with stroke and caregiving, most interviewees appeared to be outwardly coping well. A recurring theme in the interviews and visual media was ‘pagtitiis’, a culturally embedded form of quiet endurance or forbearance [39, 40] defined as ‘enduring the difficulty and stress without constant effort in confronting it’ [41]. While it reflects strength and a determination to persevere independently, it may also conceal unmet needs and discourage help‐seeking.

Traditional and alternative therapies were widely used, including ‘hilot’ or massage [42] and herbal remedies. These were often viewed as more affordable and accessible than formal healthcare. Although clinical evidence remains limited [43], some studies suggest that integrating practices such as acupuncture and herbal medicine with conventional stroke rehabilitation may improve outcomes [44]. Participants perceived these therapies as effective and empowering, potentially offering psychological benefits by enhancing a sense of control and resilience.

Faith was also a key source of strength, with religious practices commonly used as coping strategies, a pattern consistent with Filipino cultural norms [41, 45]. Religiosity has been linked to higher health‐related quality of life in Filipino populations [46], and a recent systematic review found that spirituality can reduce anxiety and depression while enhancing quality of life in stroke survivors and their caregivers [47]. The cultural concept of ‘bahala na’—entrusting one's fate to God [45]—may be associated with fatalism and dependence in illness contexts, yet it also reflects a form of adaptive coping grounded in hope, self‐efficacy, optimism, courage and spiritual meaning, signifying acceptance of uncertainty while continuing to persevere [48].

#### 4.1.3 Limited Knowledge and Awareness of Stroke and Rehabilitation

Low levels of stroke knowledge and awareness were evident. Misinformation, reliance on instinct and use of unreliable sources of information (such as social media) were common. Many participants did not understand the role of rehabilitation in recovery. These findings are apparent in other low‐income communities; for example, a focus group study in rural South Africa involving people with stroke, family caregivers and community health workers found minimal stroke knowledge, limited formal training and a reliance on self‐learning, with ‘Figuring it out by yourself’ emerging as a key theme [28]. Both people with stroke and household carers expressed a clear need for practical, accessible education and training.

#### 4.1.4 Stroke Care and Rehabilitation Needs, Challenges and Inequities, and Recommendations

The interviews revealed low availability of stroke care and rehabilitation services. Although this was a widespread issue, access to care varied, with inequities primarily associated with geographical and financial factors. Such inequities are commonly reported in LMICs [4, 5, 6] and consistent with literature on stroke care systems in the Philippines, which has documented persistent shortages of medical resources and healthcare personnel in rural areas relative to urban centres [11, 49, 50]. Despite the implementation of UHC, out‐of‐pocket expenses remain high, exacerbating difficulties in accessing stroke care [51, 52].

Despite initial reticence, the participants proposed several improvements to stroke services in the Philippines. These included increased financial assistance for medical expenses; greater provision of local services, medications and mobility aids; improved transport to healthcare facilities; more home visits by healthcare professionals; stroke‐specific support groups for people with stroke and their carers; and livelihood and return‐to‐work initiatives. These suggestions align with national recommendations [11, 50] and reflect strategies used in other LMICs, such as structured return‐to‐work programmes for people with stroke in Nigeria [53, 54].

#### 4.1.5 Implications for Policy and Practice

For policy and practice, the findings indicate the need for holistic support that addresses the physical, psychological, emotional, social and financial impacts of stroke. Current stroke policies and professional guidelines in the Philippines focus predominantly on acute care [55, 56]; there is a clear need to extend these frameworks to encompass long‐term care, rehabilitation and community reintegration when patients return home. Return‐to‐work programmes, psychosocial support and services specifically for carers should be integral components of a comprehensive stroke care strategy. Social protection mechanisms under Universal Health Care (UHC) should be strengthened and expanded to cover a wider range of stroke‐related services and costs [51, 52]. Enhanced community‐based support and opportunities for social connection are also needed for households affected by stroke; although some local support groups exist for other chronic conditions (e.g., diabetes), there is a lack of stroke‐specific support groups [57]. The findings highlight the importance of culturally relevant policies and programmes that build on local strengths while bridging gaps in formal care. Clear, evidence‐based guidance on traditional therapies—and how they may complement rather than replace modern medicine—should be provided [43]. Holistic rehabilitation programmes should incorporate innovative and culturally grounded coping strategies, including spirituality. Gendered caregiving norms must be considered when designing carer support initiatives; women may need tailored support, particularly as they may be at higher risk of caregiver burden and its negative impacts [6, 58]. To ensure equitable care, policies must ensure support for those without family or community networks.

Improving knowledge and health literacy is essential for increasing self‐efficacy and empowering people with chronic conditions to manage their own health [59, 60], which in turn can lead to better clinical outcomes [61]. Educational programmes are needed for people with stroke, household carers, BHWs and other healthcare professionals. These should cover risk factors, symptoms, rehabilitation pathways and self‐management strategies. Delivery should be flexible, multi‐format and tailored to individual needs, preferences and literacy levels [62]. The Stroke Society of the Philippines and the World Stroke Organization introduced a national stroke training programme for healthcare professionals in 2015 [49]. However, stroke awareness among community health workers, stroke survivors and their families remains limited [11]. Evidence from other LMICs demonstrates that community‐based, culturally tailored education initiatives can be effective in improving stroke literacy and reducing preferences for traditional therapies [63, 64]. Moreover, educational interventions have been shown to reduce stigma associated with stroke, which is prevalent in many LMICs and may negatively impact mental health and quality of life [65].

Strengthening health systems and implementing and evaluating programmes at the local level will be vital to addressing unmet needs and reducing inequities. The Hub‐and‐Spoke Stroke Care System, introduced in 2021, in which primary care facilities are connected to specialised stroke centres, has improved access to acute stroke care in underserved areas in the Philippines [66]. However, long‐term, home‐ and community‐based rehabilitation remains limited. Community‐Based Inclusive Development (CBID) has been initiated in selected areas to bring rehabilitation, health promotion and empowerment services closer to people with stroke, but broader Local Government Unit (LGU) level policies, funding and workforce support are needed for sustainable implementation [67, 68, 69]. Digital technologies, including smartphone apps, telemedicine, virtual reality, robotics and wearables, can be utilised to facilitate home‐based rehabilitation [70] and reduce caregiver burden [6]. The 2023 World Stroke Organization–Lancet Neurology Commission highlights these priorities for LMICs, including improved access to rehabilitation, digital health investment, integration with non‐communicable disease policies, and expanded roles for community health workers [71].

Engaging a diverse range of stakeholders is essential, with people with stroke and their carers placed at the centre of the process [5], to ensure that interventions are relevant, accessible and acceptable to those they are intended to support, while also being effective, sustainable and scalable. The Filipino ethos of ‘Bayanihan’, which emphasises communal unity and mutual support, provides a strong foundation for co‐designing locally appropriate, community‐driven solutions. Insights from this study will be triangulated with data from the broader TULAY project (including surveys, workshops and stakeholder consultations), which will inform the ongoing co‐design of self‐management and training resources aimed at improving quality of life for people with stroke and their household carers.

### 4.2 Strengths and Limitations

Strengths of this study include the use of rich, in‐depth interviews enhanced by visual elicitation and a diverse sample representing multiple regions of the Philippines. The consistency of themes across regions supports the transferability of findings. Interviews were transcribed and translated by the same researchers who conducted them, ensuring that contextual nuances, local expressions and nonverbal cues were preserved, while enabling clarification of ambiguous responses and promoting reflexivity to strengthen analytical rigour. Support from experienced qualitative researchers in the United Kingdom provided a valuable independent external perspective.

There are some methodological limitations. Potential selection bias and unexamined cultural or regional factors may limit broader applicability. The number of participants opting to share a photograph or video might have increased if the research team had provided cameras or smartphones. Although the time since stroke was captured, quantitative data on stroke severity (e.g., the National Institutes of Health Stroke Scale, NIHSS [72]) were not collected. Finally, people with stroke and their household carers were together for most interviews. While this reflected participants' preferences and cultural norms, some may have withheld personal or emotionally sensitive reflections to protect their loved ones or maintain an image of resilience. Individual interviews might have offered a more private space for disclosure.

## 5 Conclusion

This study reveals the profound impact of stroke on individuals and household carers in the Philippines. Despite limited knowledge of stroke and scarce rehabilitation services, particularly in rural, remote and low‐income areas, participants demonstrated resilience, adaptability and innovation. The findings emphasise the urgent need for culturally appropriate, community‐ and home‐based rehabilitation, alongside improved stroke education, financial assistance and community support. Future research should explore regional differences and assess the implementation and scalability of interventions. Mixed‐methods evaluations of feasibility, acceptability and effectiveness using participatory approaches will be critical for addressing inequities, supporting recovery and promoting long‐term well‐being.

## Author Contributions

**Sarah Ann Buckingham:** conceptualisation, data curation, formal analysis, methodology, supervision, validation, visualisation, writing – original draft, writing – review and editing, data analysis, data interpretation. **Maria Mercedes Barba:** conceptualisation, methodology, writing – review and editing. **Lorraine Faeldon:** conceptualisation, methodology, writing – review and editing. **Nena Marie Santos:** formal analysis, investigation, validation, writing – review and editing, data analysis, data interpretation, interview conduction (with the wider TULAY team). **Paula Melizza Valera:** formal analysis, investigation, writing – review and editing, data analysis, data interpretation, interview conduction (with the wider TULAY team). **Elda Anota:** conceptualisation, methodology, writing – review and editing. **Vergil Boac:** writing – review and editing, data analysis, data interpretation, interview conduction (with the wider TULAY team). **Myrna Estrada:** conceptualisation, methodology, writing – review and editing. **Bridie Kent:** conceptualisation, methodology, writing – review and editing. **Fiona Leggat:** conceptualisation, writing – review and editing, data analysis, data interpretation. **Roy Francis Navea:** writing – review and editing. **Aira Patrice Rueda Ong:** writing – review and editing, data analysis, data interpretation. **Nena Marie Santos:** writing – review and editing. **Annah Teves:** writing – review and editing, data analysis, data interpretation, interview conduction (with the wider TULAY team). **Fiona Jones:** conceptualisation, methodology, writing – review and editing.

## Disclosure

The views expressed in this publication are those of the authors and not necessarily those of the NIHR or the UK Government.

## Ethics Statement

Ethical approval was granted by the Philippines Department of Health Single Joint Research Ethics Board (SJREB) (Ref: SJREB‐2023‐85) and the University of Plymouth Faculty of Health Research Ethics and Integrity Committee (Ref: 2024‐4703‐5965).

## Consent

Informed consent was obtained from all participants prior to interviews.

## Conflicts of Interest

The authors declare no conflicts of interest.

## Supporting information


**Supplementary File 1:** COREQ Checklist.

**Supplementary File 2:** Interview topic guide: People with stroke (English version).

**Supplementary File 3:** Interview topic guide: Household carers (English version).

## Data Availability

The data that support the findings of this study are available from the corresponding author upon reasonable request. Due to privacy and ethical considerations, only anonymised excerpts of interview data can be shared. Full transcripts and complete datasets are not publicly available.
